# 

**Published:** 1837-07-01

**Authors:** 


### CONTENTS

OF THE

### MEDICO-CHIRURGICAL REVIEW,

### No. LIII. JULY 1, 1837,

[BEING No. XIII. OF A DECENNIAL SERIES.]

### REVIEWS.

I.

RECENT PATHOLOGICAL INVESTIGATIONS.

1. Medico-Chirurgical Transactions. Volume the Twentieth.. .. .. .. .. ..
2. Guy's Hospital Reports, No. IV. .. .. .. .. .. .. .. .. .. .. .. .. .. 1
3. The Transactions of the Provincial Medical and Surgical Association, Vol. V. 1837
4. Outlines of Human Pathology. By HERBERT MAYO, F.R.S. .. .. .. .. ..

1. Affections of the Bones .. .. .. .. .. .. .. .. .. .. .. .. .. 3
   - A. An Inquiry respecting the Process of Reparation after simple Fracture of Bones. By Mr. Bransby Cooper .. .. .. .. .. .. .. .. .. 3
   - B. Observations on some of the Forms of Atrophy of Bone. By T. Blizard Curling, Esq. .. .. .. .. .. .. .. .. .. .. .. .. .. 6
   - C. Observations on some Tumors of the Mouth and Jaws. By R. Liston, Esq. .. .. .. .. .. .. .. .. .. .. .. .. .. .. .. 10
2. On Inflammation, Chronic Disease, and Perforative Ulceration of the Cæcum, &c. By John Burne, M.D.. .. .. .. .. .. .. .. .. .. .. .. 19
3. Affections of the Heart and Arteries.
   - A. Remarkable Case of Varicose Aneurysm. By Mr. J. G. Perry .. .. 24
   - B. Remarks on two Forms of Atrophy of the Heart's Valves, &c. By Dr. Kingston .. .. .. .. .. .. .. .. .. .. .. .. .. .. .. 27
4. Affections of the Lungs, &c.
   - A. Case of Diaphragmatic Hernia. By Dr. W. Norris .. .. .. .. .. 30
   - B. Dissection of a Case in which one Lung had been compressed by Fluid Effusion .. .. .. .. .. .. .. .. .. .. .. .. .. .. .. .. 31
   - C. On the Pathology of Pulmonary Tubercles. By Dr. Kingston .. .. 32
   - D. Observations on the Diagnosis of Pneumonia. By Dr. Addison .. .. 37
   - E. On Black Expectoration, and the Deposition of Black Matter in the Lungs. By Dr. William Thomson .. .. .. .. .. .. .. .. .. .. .. .. 41
5. Ovarian Tumour successfully removed By Mr. Jeaffreson .. .. .. .. 47
6. Case of Tetanus successfully treated by Subcarbonate of Iron. By J. Hamerton, Esq. .. .. .. .. .. .. .. .. .. .. .. .. .. .. 48

II.

Elements of Medicine, Vol. I. On Morbid Poisons. By ROBERT WILLIAMS, M.D. .. 49

1. Typhoid Poison .. .. .. .. .. .. .. .. .. .. .. .. .. .. .. 49

2. On the Poison of Scarlatina .. .. .. .. .. .. .. .. .. .. .. 59

   - A. Scarlatina Gravior .. .. .. .. .. .. .. .. .. .. .. .. .. 61

III.

Lectures illustrative of certain local Nervous Affections. By Sir BENJAMIN C. BRODIE, Bart. F.R.S.. .. .. .. .. .. .. .. .. .. .. .. .. .. .. .. 68

1. Pathology of Hysteria .. .. .. .. .. .. .. .. .. .. .. .. .. 69

IV.

The Economy of Health, or the Stream of Human Life from the Cradle to the Grave, &c. By J. Johnson, M.D. .. .. .. .. .. .. .. .. .. .. .. .. .. .. 79

V.

Philosophical Anatomy.

1. Philosophie Anatomique. Par M. le Chev. Geoffroy Saint Hilaire .. ..
2. Histoire des Anomalies de l'Organization, &c. Par M. Isidore Geoffroy St. Hilaire .. .. .. .. .. .. .. .. .. .. .. .. .. .. .. .. .. .. 83
3. Sketch of the comparative Anatomy of the Nervous System, with Remarks on its Development in the Human Embryo. By John Anderson, M. E. S. .. ..

VI.

Cyclopædiæ and Dictionaries of Medicine and Surgery.

1. A Dictionary of Practical Medicine, &c. By James Copland, M.D. .. .. ..
2. The American Cyclopædia of Practical Medicine and Surgery, &c. Edited by Isaac Hayes, M.D. .. .. .. .. .. .. .. .. .. .. .. .. .. .. .. .. .. 129
3. The Cyclopædia of Practical Surgery, comprising a Series of Original Dissertations on Operative Medicine. Edited by W. Costello, M.D. .. .. .. .. .. ..
   - A. Deafness from Affections of the External Ear .. .. .. .. .. .. .. .. 130
   - B. Deafness from Disease of the Eustachian Tube .. .. .. .. .. .. .. .. 132
   - C. Deafness from Affections of the Auditory Nerves .. .. .. .. .. .. .. 134
   - D. Deafness and Dumbness.. .. .. .. .. .. .. .. .. .. .. .. .. .. 136
   - E. Alimentary Concretions.. .. .. .. .. .. .. .. .. .. .. .. .. .. 138

### PERISCOPE.

### Bibliographical Notices.

1. The British Flora Medica, or History of the Medicinal Plants of Great Britain. By B. H. Barton, F.L.S. .. .. .. .. .. .. .. .. .. .. .. .. .. .. .. 145
2. Mr. Headland's Oration before the London Medical Society, 1837 .. .. .. .. .. 150
3. A Treatise on the Prevention and Cure of Pulmonary Consumption. By R. Little, M.D. 151
4. Philosophy of Living.
   1. The Philosophy of Living. By Caleb Ticknor, M.D. .. .. .. .. .. .. 152
   2. The Philosophy of Living. By Herbert Mayo, F.R.S. .. .. .. .. .. .. 152
5. The Works of John Hunter F.R.S., with Notes. By James F. Palmer, Esq... .. 153
6. New Works on Anatomy and Physiology.
   1. Cyclopædia of Anatomy and Physiology. Part X.. .. .. .. .. .. .. .. 154
   2. Nouvelles Recherches sur la Structure de la Peau. Par M. Breschet .. .. 155
   3. Anatomie du Système Dentaire. Par Ph. Blandin, &c. .. .. .. .. .. .. 155
   4. Lecons d'Anatomie Comparée de George Cuvier .. .. .. .. .. .. .. .. 155
   5. Anatomie Descriptive. Par J. Cruveilhier .. .. .. .. .. .. .. .. .. 155
7. Recent Anatomical Plates
   1. Quain's Plates .. .. .. .. .. .. .. .. .. .. .. .. .. .. .. .. .. 156
   2. Bourgery's Plates .. .. .. .. .. .. .. .. .. .. .. .. .. .. .. .. 156
8. Recent Works on Diseases of the Skin.
   1. A Practical Treatise on Diseases of the Skin, &c. By Samuel Plumbe, &c. .. 156
   2. A Practical Compendium of the Diseases of the Skin, &c. By Jonathan Green, M.D. .. .. .. .. .. .. .. .. .. .. .. .. .. .. .. .. .. 158
9. An Address to the Worcestershire Natural History Society. By C. Hastings, M.D. 158
10. An Examination of Phrenology. By T. Sewall, M.D. .. .. .. .. .. .. .. 159

### Spirit of the English Periodicals, and Notices of English Medical Literature.

1. Singular Case of Periodical Monomania. By James Johnson, M.D. .. .. .. .. 161
2. Effects of Solitary Confinement on the Health of Soldiers in Hot Climates. By Mr. Malcolmson .. .. .. .. .. .. .. .. .. .. .. .. .. .. .. .. .. .. 170
3. Nitrate of Silver in immense Doses .. .. .. .. .. .. .. .. .. .. .. .. 172
4. On the Effects of Cold. By Sir Henry Halford .. .. .. .. .. .. .. .. .. 172
5. Retroversio Uteri .. .. .. .. .. .. .. .. .. .. .. .. .. .. .. .. .. 173
6. Dr. Dewins on Colchicum .. .. .. .. .. .. .. .. .. .. .. .. .. .. .. 174

7. Malignant Disease of the Stomach, with remarkable Symptoms .. .. .. .. .. 176
8. Sir Francis Smith on Creosote as an External Application .. .. .. .. .. 177
9. Dr. Lendrick on the Use of the Nitro-muriatic Acid Bath .. .. .. .. .. .. 179
10. Dr. Fingerhuth on Hypertrophy of the Mammary Gland.. .. .. .. .. .. 179
11. Thoracic Inflammation masked .. .. .. .. .. .. .. .. .. .. .. .. 181
12. Use of the solid Nitrate of Silver for the Gonorrhœa of Women .. .. .. .. 182
13. On the Condition of the Brain in Sleep, Epilepsy, &c. By Herbert Mayo .. .. 183
14. Vital Statistics.
1. Value of Life in Geneva .. .. .. .. .. .. .. .. .. .. .. .. 184
2. Abuses of Vital Statistics .. .. .. .. .. .. .. .. .. .. .. .. 185
15. The Bruit du Diable .. .. .. .. .. .. .. .. .. .. .. .. .. 186
16. Chemical Constituents of Calamine .. .. .. .. .. .. .. .. .. .. 187
17. Seven Half-crowns swallowed, and voided after 20 months .. .. .. .. .. 187
18. Cough ending in Emphysema of the Body .. .. .. .. .. .. .. .. .. 188
19. Diabetes.
1. On the comparative State of Urea in the healthy and diseased Urine. By R. M'Gregor, Esq. .. .. .. .. .. .. .. .. .. .. .. .. 188
2. Case of Diabetes Mellitus. By Henry Snowden, Esq. .. .. .. .. .. .. 188
3. Mr. Leach on Lytta in Diabetes .. .. .. .. .. .. .. .. .. .. 190
20. The Northern Flora. By Alexander Murray, M.D.. .. .. .. .. .. .. 190
21. Tic Douloureux relieved by Galvanism .. .. .. .. .. .. .. .. .. 190
22. Substance of a Statistical Report from the Morgue, 1836 .. .. .. .. .. 191
23. Varieties of the Lobelia Inflata dependent on Soil .. .. .. .. .. .. .. 192
24. On Wounds of the Heart. By Mr. Lees .. .. .. .. .. .. .. .. .. 193
25. Transatlantic Snuff-chewing .. .. .. .. .. .. .. .. .. .. .. 196
26. Medicine in India .. .. .. .. .. .. .. .. .. .. .. .. .. 196
27. Remarkable Case of Cephalœa .. .. .. .. .. .. .. .. .. .. .. 200

### Spirit of the Foreign Periodicals, &c.

1. Biographical Sketch of the late Baron Dupuytren .. .. .. .. .. .. .. 201
2. Biographical Sketch of the late Baron Desgenettes .. .. .. .. .. .. .. 204
3. Inflammation of the Spinal Marrow—Alleged frequency of this Disease in Agues, &c. 206
4. Extracts from, and remarks on, M. Bouillaud's Clinique .. .. .. .. .. .. 209
A. Typhus Fever .. .. .. .. .. .. .. .. .. .. .. .. .. 209
B. Intermittent Fever and Neuralgia .. .. .. .. .. .. .. .. .. .. 209
5. Increase of Suicide in Paris, Poisoning from Colchicum, &c. .. .. .. .. .. 211
6. On Gastro-Malacia .. .. .. .. .. .. .. .. .. .. .. .. .. 213
7. Cursory Remarks on Hydrocephalus Acutus, with Cases .. .. .. .. .. .. 219
8. Cases of Croup actively and successfully treated .. .. .. .. .. .. .. 225
9. Peculiar Spasmodic Affection of the Thumb in writing, &c. .. .. .. .. .. 226
10. Idiopathic Tetanus and Trismus connected with Ramollissement of the Spinal Cord, 227
11. Case of Spina Bifida. With Remarks .. .. .. .. .. .. .. .. .. 228
12. Imperforate Anus. Successful Formation of an Artificial Anus in the Right Groin 230
13. Cases illustrative of Puerperal Fever .. .. .. .. .. .. .. .. .. .. 230
14. Lamentable Case of difficult Parturition .. .. .. .. .. .. .. .. .. 234
15. Professor Nægele on difficult Parturition from Agglutination of the Os Uteri .. 235
16. Treatment of the Insane .. .. .. .. .. .. .. .. .. .. .. .. 238
17. Professor Lallemand on Spermatorrhea .. .. .. .. .. .. .. .. .. 239
18. On the Curative Properties of Chlorine, and of the Chloride of Lime .. .. .. 240

### Clinical Review, and Hospital Reports.

1. Hospital Medicine and Hospital Surgery .. .. .. .. .. .. .. .. .. 241

HULL GENERAL INFIRMARY.

2. Report of some Cases by Mr. Henry Snowden .. .. .. .. .. .. .. .. 243
A. Softening of the Brain .. .. .. .. .. .. .. .. .. .. .. .. 243
B. Cases of Adhesion of the Pericardium .. .. .. .. .. .. .. .. .. 244
C. Tubercular Sycosis of the Legs .. .. .. .. .. .. .. .. .. .. 245

DONCASTER DEAF AND DUMB ASSOCIATION.

3. Differences in the Power of acquiring the Language of Gesture in the Deaf and Dumb .. .. .. .. .. .. .. .. .. .. .. .. .. .. .. 245

HOSPITAL OF THE 2D DRAGOON GUARDS.

4. Supposed Glanders in the Human Subject .. .. .. .. .. .. .. .. .. .. 246

NORTH LONDON HOSPITAL.

5. Dislocation of the Carpus backwards .. .. .. .. .. .. .. .. .. .. .. .. 249
6. Guy's Hospital Reports, No. 4. .. .. .. .. .. .. .. .. .. .. .. .. .. 249
   1. A Practical View of Lithotrity. By Mr. Aston Key .. .. .. .. .. .. 250
      A. Dangers of Lithotomy .. .. .. .. .. .. .. .. .. .. .. .. .. 254
      B. Lithotrity.. .. .. .. .. .. .. .. .. .. .. .. .. .. .. .. .. 258
   2. Two Cases of Poisoning by Arsenious Acid. By Mr. A. S. Taylor .. .. 263
   3. On the Safety-Valve of the Heart .. .. .. .. .. .. .. .. .. .. .. 265
   4. Reparation of Fractured Bones .. .. .. .. .. .. .. .. .. .. .. .. 265
   5. Cases and Observations on the Diagnosis of Tumors at the Base of the Brain 265
      A. Tumors at the Basis Cranii .. .. .. .. .. .. .. .. .. .. .. .. 265
      B. Lesions of the Spinal Cord.. .. .. .. .. .. .. .. .. .. .. .. 270
      C. Lesions of the Brain .. .. .. .. .. .. .. .. .. .. .. .. .. .. 271

ROYAL NAVAL HOSPITAL PLYMOUTH.

7. Case of Gastro-Enteritis, and Phlebitis Portalis. By Sir David Dickson .. .. 273

BIRMINGHAM EYE INFIRMARY.

8. Report of the Cases attended by R. Middlemore, Esq. ., .. .. .. .. .. .. 276
   1. Irritable Ophthalmia .. .. .. .. .. .. .. .. .. .. .. .. .. .. .. 276
   2. Carcinomatous Growth from the Cornea and Sclerotica .. .. .. .. .. 276
   3. Melanotic Growth from the Semi-lunar Membrane .. .. .. .. .. .. 277
   4. Ossification of the Cornea.. .. .. .. .. .. .. .. .. .. .. .. .. 277
   5. Circumscribed Vascular Prominence of the Cornea .. .. .. .. .. .. 277
   6. Extensive Bulging of the Sclerotica .. .. .. .. .. .. .. .. .. .. 278
   7. Disease resembling malignant Affection of the Eye-ball.. .. .. .. .. 278

WORCESTER INFIRMARY.

9. Report for the Years 1835 and 1836 .. .. .. .. .. .. .. .. .. .. .. .. 278
10. Clinical Facts and Observations .. .. .. .. .. .. .. .. .. .. .. .. .. 280
   1. Opening a small Abscess .. .. .. .. .. .. .. .. .. .. .. .. .. .. 280
   2. To prevent Scars in the Neck .. .. .. .. .. .. .. .. .. .. .. .. 280
   3. How to open a large Abscess .. .. .. .. .. .. .. .. .. .. .. .. 281
   4. How to open a deep Abscess in the Neck.. .. .. .. .. .. .. .. .. 281
   5. Uncertainty of dividing the Stricture in Hernia, external to the Sac .. .. 281
   6. Why the Recurrent Nerve is recurrent .. .. .. .. .. .. .. .. .. .. 282
   7. Cause of the Inclusion of the Tendon of the Biceps in the Scapulo-humeral Capsule .. .. .. .. .. .. .. .. .. .. .. .. .. .. .. .. .. .. 282
   8. Expense of Patients in the Royal Infirmary of Glasgow .. .. .. .. .. .. 282
   9. Gouty Ramollissement of the Spinal Marrow .. .. .. .. .. .. .. .. 283
   10. Reformation of Factory Morals.. .. .. .. .. .. .. .. .. .. .. .. 284
   11. Successful Treatment of Lead-Colic .. .. .. .. .. .. .. .. .. .. 284
   12. Treatment of Pleurodynia .. .. .. .. .. .. .. .. .. .. .. .. .. 285
   13. Prevalence of Tape-Worm in Birmingham .. .. .. .. .. .. .. .. .. 285

### Miscellanies.

1. Irish Medical Charities' Bill .. .. .. .. .. .. .. .. .. .. .. .. .. .. 285

OBITUARY.

1. General Bill of Mortality for London, from Dec. 1835, to Dec. 15, 1836 .. .. 287
2. Death of Dr. Mower .. .. .. .. .. .. .. .. .. .. .. .. .. .. .. .. 287
3. Death of Professor Himly .. .. .. .. .. .. .. .. .. .. .. .. .. .. 288
4. Death of Dr. Cummin .. .. .. .. .. .. .. .. .. .. .. .. .. .. .. 288
5. Death of M. Dubois .. .. .. .. .. .. .. .. .. .. .. .. .. .. .. 288
6. Death of Mr. Vance .. .. .. .. .. .. .. .. .. .. .. .. .. .. .. 288

### Extra-Limites.

1. Mr. Annesley's Reply to the late Mr. Twining .. .. .. .. .. .. .. .. .. 289
2. London Missionary Society .. .. .. .. .. .. .. .. .. .. .. .. .. .. 290
3. General Registration of Diseases.. .. .. .. .. .. .. .. .. .. .. .. 291
4. Case of Empyema cured by Paracentesis. By Dr. Edward, of Forfar .. .. .. .. 292
5. Principal Original Papers published in the Weekly and Monthly British Journals, between 25th March and 10th June, 1837 .. .. .. .. .. .. .. .. .. 293

BIBLIOGRAPHICAL RECORD .. .. .. .. .. .. .. .. .. .. .. .. .. .. .. .. 295

